# Wide Variability in Emergency Physician Admission Rates: A Target to Reduce Costs Without Compromising Quality

**DOI:** 10.5811/westjem.2016.7.30832

**Published:** 2016-08-08

**Authors:** Jeffrey J. Guterman, Scott R. Lundberg, Geoffrey P. Scheib, Sandra G. Gross-Schulman, Mark J. Richman, Chien-Ju Wang, David A. Talan

**Affiliations:** * David Geffen School of Medicine at UCLA, Department of Medicine, Los Angeles, California; † Olive View-UCLA Medical Center, Department of Emergency Medicine, Sylmar, California; ‡ Los Angeles County Department of Health Services, Los Angeles, California; § Northwell Health Long Island Jewish Medical Center, Department of Emergency Medicine, New Hyde Park, New York; ¶ Hofstra University School of Medicine, Department of Emergency Medicine, Hempstead, New York; || Los Angeles County Department of Public Health, Los Angeles, California

## Abstract

**Introduction:**

Attending physician judgment is the traditional standard of care for emergency department (ED) admission decisions. The extent to which variability in admission decisions affect cost and quality is not well understood. We sought to determine the impact of variability in admission decisions on cost and quality**.**

**Methods:**

We performed a retrospective observational study of patients presenting to a university-affiliated, urban ED from October 1, 2007, through September 30, 2008. The main outcome measures were admission rate, fiscal indicators (Medicaid-denied payment days), and quality indicators (15- and 30-day ED returns; delayed hospital admissions). We asked each Attending to estimate their inpatient admission rate and correlated their personal assessment with actual admission rates.

**Results:**

Admission rates, even after adjusting for known confounders, were highly variable (15.2%–32.0%) and correlated with Medicaid denied-payment day rates (p=0.038). There was no correlation with quality outcome measures (30-day ED return or delayed hospital admission). There was no significant correlation between actual and self-described admission rate; the range of mis-estimation was 0% to 117%.

**Conclusion:**

Emergency medicine attending admission rates at this institution are highly variable, unexplained by known confounding variables, and unrelated to quality of care, as measured by 30-day ED return or delayed hospital admission. Admission optimization represents an important untapped potential for cost reduction through avoidable hospitalizations, with no apparent adverse effects on quality.

## INTRODUCTION

Healthcare costs are at the forefront of discussions of the United States healthcare system.^1^ The U.S. spends more than any other country on medical care^2^ without demonstrable differential in positive outcomes for many conditions.^3^ Inpatient hospitalizations drive one-third of total healthcare expenditure in the U.S.^4^,^5^ Forty percent of all admissions,^3^ and as many as 70% of admissions for major service lines such as general medicine, pulmonary, gastrointestinal, general surgery, and orthopedics^6^ originate in the emergency department (ED). Publicly supported EDs and uninsured patients have higher ED admission rates than private sector hospitals.^7^

Emergency physicians’ decisions to admit rely on assorted cues and information from patients. This framework^4^,^5^ is the basis for most clinical decision-making today. Objective criteria and scoring systems have been proposed to supplant this subjective model.^8^,^9^ However, attending physician judgment remains the standard of care for ED admission decisions. Inter-physician variability in admission decisions and its impact on cost and quality have not been studied in detail. In addition to reducing costs, avoiding unnecessary admissions reduces patient exposure to high-risk patient safety events such as venous thromboembolism (VTE),^10^ central line-associated blood stream infections (CLABSI),^11^ catheter-associated urinary tract infections (CAUTI),^12^ falls,^13^ and medication errors.^14^

Observations by the authors led us to suspect there would be significant differences between emergency physicians in their admission rates at our institution. We sought to understand these admission patterns and their effects on financial and quality indicators. We hypothesized a positive correlation between admission rates and inappropriate admissions, and a negative correlation between admission rates and repeat ED visits or delayed hospital admission (as such patients were admitted at the index visit).

As a proxy for inappropriate admissions, we used rates of Medicaid (Medi-Cal) denied-payment days. To ensure appropriate admissions, Medicaid (“Medi-Cal” in California) reviews admission documentation and may deny payment for admission on the basis of inadequate documentation, delays in care, and lack of medical necessity for admission.^15^ We analyzed the relationship between rates of inpatient admission, quality indicators (15- and 30-day return visits, delayed hospital admissions), and insurance denial of reimbursement for inpatient days. Variability not explained by known risk factors for admission might suggest other means of decreasing admission rates, thereby controlling costs.

## METHODS

This retrospective, observational study was conducted at a 377-licensed bed, publicly supported, academic teaching hospital. It is staffed to 200 beds and serves a medically-indigent population, without cardiothoracic, neurosurgical, or inpatient orthopedic services. During the study period, the ED had 23 beds and provided over 41,000 visits annually. While not a trauma center, it is one of two principal training sites for an emergency medicine (EM) residency training program. Patients are evaluated and cared for by EM, internal medicine, or family practice residents, with EM attending supervision. Patient visits are associated with each treating (resident) and supervising (attending) provider. Non-emergent patients triaged during daytime hours are seen in separate urgent care clinics staffed by general internists and pediatricians. Patients seen in non-emergent areas were excluded from this study.

Around-the-clock EM attending coverage is provided by full- and part-time faculty board certified in EM. Many are also board certified in internal medicine. Insurance coverage of the inpatient population included 51% Medicaid, 38% uninsured, 8% Medicare, and 3% private insurance. Sixty-three percent of inpatient admissions originate in the ED. This above-average rate of ED admissions is consistent with public hospitals serving medically-indigent populations.^7^

All ED encounters from October 1, 2007, through September 30, 2008, were included in the study. Those admitted to the hospital were associated with the ED attending physician at the time the decision to admit was made. Attending physicians with fewer than 100 admissions during the study period were excluded from analysis to reduce spurious results from small-number analysis. Study physicians covered the ED more than 85% of the time. The study population is depicted graphically in the figure.

This study used data contained in the Advanced Triage and Emergency Medicine Management (ATEMM) system. ATEMM is a fully-computerized operational workflow program providing and collecting real-time point-of-care ED information. No separate data collection was required for data elements related to patient-specific activity in the ED.

We obtained utilization review (UR) data for Medicaid patients from an electronic system used by the medical director for utilization review. Data on board certification status and practice duration were gleaned from medical staff records. The attending schedule was abstracted from the AmIOn scheduling system (Spiral Software: Norwich, VT).

For each hospital admission, the UR database was cross-referenced to identify Medicaid beneficiaries**.** The UR database has a framework for categorization of Medicaid denied-payment days. One categorization is “physician-attributable denied days,” which refers to days whose payment denial was related to inappropriate admission or inadequate documentation, rather than to administrative reasons such as difficulty transferring to a higher level of service. We classified admissions with one or fewer approved days as a proxy for a denied admission. Patients admitted, only to be sent home by the inpatient admission team the same day, were counted as one denied day. We analyzed physician-attributable and non-attributable (e.g., awaiting transfer, placement, or administrative actions) denied days separately. Each attending physician’s admission rate was correlated with the percentage of admissions with at least one denied day and with quality indicators (ED repeat visits and delayed hospitalizations within 15 and 30 days of the index ED discharge).

We defined ED repeat visits as a return to our ED or urgent care clinic for any reason within 15 and 30 days following an index presentation. Delayed hospitalizations were defined as hospitalization at our institution for any reason (via the ED or not) within 30 days of an index presentation for which the patient was discharged from the ED.

We analyzed data for known confounders of admission rate: distribution of attending shifts in the day, evening, or night; percentage of pediatric patients; distribution of patient arrivals in the day, evening, or night; percentage of patients who arrived by ambulance,^16^ and suspected confounders of admission rate: number of years as an EM attending; and full- or part-time faculty status. Finally, we asked each attending to estimate their inpatient admission rate and correlated their personal assessment with actual admission rates.

Statistics were performed using SPSS 11.5 (Chicago, IL) and Microsoft Excel 2003 Data Analysis Tool Pack (Redmond, WA). For all analyses, p <0.05 was considered significant.

The study was approved by the institutional review board.

## RESULTS

A total of 41,248 ED visits occurred during the study timeframe; 31,373 (76.1%) were discharged, 8,813 (21.4%) were admitted, and 1,062 (2.6%) had another disposition (e.g., left against medical advice, transferred, etc.). Eight thousand eighty-eight patients (19.6%) were admitted by 20 attending physicians. Nine attending physicians were full time; 12 had additional board certification, all in internal medicine. Attendings had practiced between one and 27 years. Individual physician shift distribution ranged from 2.8% to 68.1% day shifts, 31.4% to 87% evening shifts, and 0% to 58.6% night shifts. Comparing demographic characteristics of patients seen by various attendings, the range of mean ages, percent who were male, and percent who arrived by ambulance of all patients seen by individual attending physicians was 37 to 43 years, 46.8% to 52.2%, and 5.9% to 7.8%, respectively.

Results are summarized in the table. Admission rates by attending ranged from 15.2% to 32.0%. Physician-attributable Medicaid denied days by attending ranged from 0% to 14.5%. There remained a significant, positive correlation (p = 0.038) between admission rate and percent of patients with at least one denied day after multivariate adjustment for distribution of attending shifts and patient arrival times, percentage of pediatric and ambulance-arrival patients, number of years as an EM attending, and full- or part-time faculty status. There was no correlation between admission rate and total number of denied days.

Returns to the ED after initial ED discharge ranged from 8.0% to 13.5% within 15 days and 12.5% to 19.1% within 30 days. Delayed inpatient admissions ranged from 1.2% to 3.6% within 15 days and 2.0% to 5.6% within 30 days of an index presentation for which the patient was discharged from the ED. The correlations between admission rate and 15-day and 30-day ED returns, and between admission rate and 15-day and 30-day delayed admission, were not significant.

Attending physician estimates of their admission rate ranged from 7% to 33%. Seventy-one percent overestimated and 24% underestimated their actual admission rate. The range of mis-estimation was 0% to 117%. Forty-eight percent estimated their admission rate within 20% of their actual admission rate; 24% were between 20 and 50% of their actual rate; 29% of the estimates were beyond 50%. There was no significant correlation between actual and self-described admission rate.

## DISCUSSION

There was more than 100% variability among ED attending physicians in their decision to admit, with admission rates of some providers greater than twice the rates of others, unexplained by known confounders. This enormous variability has important implications for healthcare cost and quality. Inpatient admissions account for $600–800 billion expenditure annually.^17^,^18^ Emergency physicians are in a key position to moderate escalating healthcare costs associated with inpatient care, as their decisions impact at least 40% of admissions.^3^ Reducing admissions by 10–25% (well below the variation observed in this study) could save approximately 1.0–2.5% of total health expenditures.^19^ Additional benefit could be achieved through avoiding high-risk events such as VTE, CLABSI, CAUTI, falls, and medication errors. The potential economic and patient care impact of optimizing physician admission decisions from the ED remains an underappreciated component of healthcare redesign. Admitting practice patterns should promote appropriate and efficient use of the healthcare delivery system without sacrificing quality of care. Greater collaboration between hospitalist physicians and greater care coordination/case management may reduce unnecessary admissions. Variable admission patterns based on differences in patient,^20^ hospital,^21^ and geographic characteristics^22^ have been shown, with consequent variation in resource expenditure.^23^ Physician-level differences have been shown to influence cost of care, for example, in the intensive care unit, where such differences were not associated with lower mortality rates.^24^ To our knowledge, this is the first demonstration of physician-level variability in adult ED admission rates and has significant implications for national healthcare cost control.

Variability in admission rate appears to be due, in part, to variation in emergency physician risk preference.^25^ If all physicians in our cohort had the same admission behaviors as the theoretical “optimal” physician (lowest admission, denied day, revisit, and delayed hospital admission rate), then, at this hospital alone, there would have been 2,518 fewer inpatient admissions, 430 fewer delayed hospitalizations, and 2,466 fewer repeat ED visits during the study period. The inpatient cost savings are clear; the corresponding increase in outpatient cost associated with non-inpatient care delivery is less well-defined.

This study found a significant positive correlation between admission rate and Medicaid denied days at this ED. The “optimal” admission rate depends on point of view. A hospital with a predominantly fee-for-service private insurance population will benefit fiscally from emergency physicians with high admission rates if those providers do not have correspondingly-high denied day rates. In a capitated integrated delivery network, ED physicians with high admission rates will adversely affect net income because additional admissions accrue costs without corresponding reimbursement.

Evidence-based ED admission decision-support systems, such as InterQual®, which have been implemented in real time in some institutions, often conflict with attending physician admission decisions; Glassman, et al. found an appropriateness rate of only 49% comparing medical admissions based on clinician discretion against InterQual® Criteria (1995).^9^ Determining admission appropriateness based on severity of illness and intensity of service criteria may not capture certain elements relevant to disposition decision-making (e.g., patient self-efficacy with home care).

There is need to improve methods of real-time or near-real-time feedback on the appropriateness of admission decisions to EM trainees and practitioners. This must not be limited to “edge cases” of dramatically-bad decisions but, rather, the differences in routine decisions we found among our high-volume, board certified attending physicians. It remains unclear what combination of subjective judgment and objective criteria are most advantageous. There remains much work to be done to determine the appropriate methodology for teaching, assessing, and providing feedback to optimize admission decision-making. Contributing to the dilemma is that many of those who teach others had little insight into their own admission rates.

## LIMITATIONS

This study has several limitations. It was conducted at a single academic site treating a medically-indigent population. Re-presentations and re-admissions were captured only from this institution. Inpatient denied days is an imperfect proxy for appropriateness of admission, as designation of days as “denied” or “covered” depends on activities beyond control of the ED physician (e.g., daily documentation of need for continued hospitalization). Emergency physician decision to admit is influenced by many factors, including the availability of consultants and timely outpatient follow up, arrival time, means of arrival and, occasionally, patient preference. It was not possible to control for all potential confounders to isolate provider variation as the singular determinant behind widely-disparate admission rates.

## CONCLUSION

Variability in emergency physician admitting patterns impacts the global cost and quality of healthcare. A mechanism to routinely track and provide feedback of admission decisions and subsequent outcomes to EM physicians in training and practice could decrease variability and produce more predictable patterns. The dramatic variability in admission rates should be confirmed in other teaching and non-teaching environments and with other payers to determine if the results can be generalized.

## Figures and Tables

**Figure:**
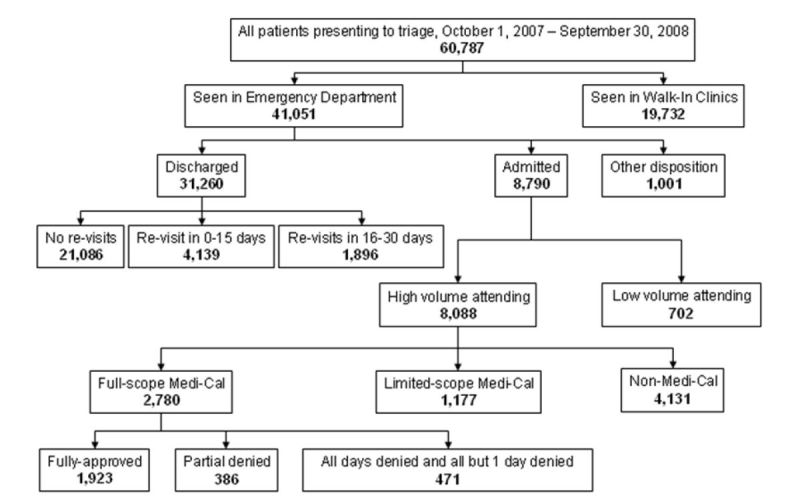
Study inclusion cohort.

**Table:** Attending physician patient profiles.

Attending alias	Admission rate (%)†	% Medicaid admissions‡	% of Medicaid admissions with 1 or more physician-attributable denied days‡	15-Day returns to ED (%)†	30-Day returns to ED (%)†	15-Day delayed admission (%)†	30-Day delayed admission (%)†	% Arrive by ambulance †	% Male gender†	Mean patient age†
MD 1	32.0	34.6	10.7	10.8	17.5	1.6	3.2	5.9	49.2	42
MD 2	30.0	32.8	9.5	12.8	18.5	2.2	4.2	6.3	48.0	42
MD 3	29.9	35.6	9.0	11.0	16.3	2.8	3.7	6.1	49.9	41
MD 4	29.1	33.9	14.5	9.2	14.1	1.8	2.2	7.8	49.4	42
MD 5	26.7	32.4	8.2	13.5	18.1	2.6	3.4	5.2	50.7	40
MD 6	26.5	41.3	6.5	10.5	14.5	1.9	2.7	5.0	46.8	43
MD 7	25.0	34.9	11.8	9.7	14.1	2.0	3.0	6.6	47.2	41
MD 8	24.0	34.4	8.6	12.1	16.9	1.2	2.0	6.5	49.7	40
MD 9	22.2	30.9	5.6	9.2	14.9	2.7	4.2	6.8	52.0	41
MD 10	21.7	30.2	12.1	11.0	15.6	1.7	2.5	5.9	48.8	41
MD 11	21.4	33.0	7.5	11.4	16.3	2.6	3.9	5.5	48.7	40
MD 12	20.6	36.6	7.7	11.5	17.7	2.2	3.4	6.0	47.6	41
MD 13	20.0	30.6	7.5	11.5	19.1	2.7	4.5	5.8	49.7	40
MD 14	19.3	36.2	9.6	8.3	13.4	1.5	2.4	6.2	48.8	40
MD 15	19.2	23.4	0.0	10.2	14.7	1.9	2.4	5.5	52.2	40
MD 16	18.7	39.0	5.5	8.0	12.5	1.6	2.8	5.5	48.1	41
MD 17	18.4	35.8	6.9	13.2	18.9	3.6	5.6	6.0	48.8	40
MD 18	18.4	35.0	11.1	11.3	15.9	3.3	4.2	5.4	50.3	40
MD 19	18.2	35.8	4.4	12.3	16.7	3.0	4.1	6.4	48.5	41
MD 20	16.3	36.1	7.2	10.3	14.7	2.7	3.6	5.1	50.3	41

Data Sources:

† Advanced Triage and Emergency Medicine Management (ATEMM),

‡ Utilization Review database.

## References

[1] Orszag PR (2009). Health costs are the real deficit threat. Wall Street Journal.

[2] Angrisano C, Farrell D, Kocher B (2007). Accounting for the Cost of Health Care in the United States.

[3] Hertzman C, Siddiqi A (2008). Tortoises 1, Hares 0: How Comparative Health Trends between Canada and the United States Support a Long-term View of Policy and Health. Health Policy.

[4] BlueCross BlueShield Association (2003). National Healthcare Trends: The Nation’s Healthcare Dollar.

[5] BlueCross Blue Shield Forces Influencing Inpatient Hospital Costs in the United States.

[6] Healthcare Financial Management (2007). The Emergency Department as Admission Source.

[7] Stern RS, Weissman JS, Epstein AM (1991). The emergency department as a pathway to admission for poor and high-cost patients. JAMA.

[8] Fine MJ, Auble TE, Yealy DM (1997). A prediction rule to identify low-risk patients with community-acquired pneumonia. New Eng J Med.

[9] Glassman PA, Lopes JH, Witt T (1997). Using proprietary methods to evaluate acute care admissions to a Veterans Affairs tertiary care center: are the appropriateness criteria appropriate?. Am J Med Qual.

[10] Cayley WE (2007). Preventing deep vein thrombosis in hospital inpatients. BMJ.

[11] CDC (2011). Vital signs: central line-associated blood stream infections—United States, 2001, 2008, and 2009. MMWR.

[12] Nicolle LE (2014). Catheter associated urinary tract infections. Antimicrob Resist Infect Control.

[13] Staggs VS, Mion LC, Shorr RI (2014). Assisted and Unassisted Falls: Different Events, Different Outcomes, Different Implications for Quality of Hospital Care. Jt Comm J Qual Saf.

[14] Radley DC, Wasserman MR, Olsho LEW (2013). Reduction in medication errors in hospitals due to adoption of computerized provider order entry systems. J Am Med Inform Assoc.

[15] Lundberg S, Balingit P, Wali S (2010). Cost-Effectiveness of a Hospitalist Service in a Public Teaching Hospital. Acad Med.

[16] Guterman JJ, Franaszek D, Murdy D (1985). The 1980 patient urgency study: Further analysis of the data. Ann Emerg Med.

[17] The Kaiser Family Foundation Health Expenditures by State of Residence, United States.

[18] American Hospital Association TrendWatch Chartbook, Chapter1: Trends in the Overall Health Care Market.

[19] Landon BE, Honigman L, Smulowitz PB (2013). A Novel Approach to Identifying Targets for Cost Reduction in the Emergency Department. Ann Emerg Med.

[20] Khaliq AA, Broyles RW (2006). Hospital admissions: who is admitted through the emergency department?. Health Serv Manage Res.

[21] Rosenthal GE, Harper DL, Shah A (1997). A Regional Evaluation of Variation in Low-Severity Hospital Admissions. J Gen Intern Med.

[22] Fisher ES, Bynum JP, Skinner JS (2009). Slowing the Growth of Health Care Costs-Lessons from Regional Variation. New Eng J Med.

[23] Wennberg JE, Barnes BA, Zubkoff M (1982). Professional Uncertainty and the Problem of Supplier-Induced Demand. Soc Sci Med.

[24] Garland A, Shaman Z, Baron J (2006). Physician-attributable differences in intensive care unit costs: a single-center study. Am J Respir Crit Care Med.

[25] Nightingale SD (2008). Risk Preference and Admitting Rates of Emergency Room Physicians. Med Care.

